# Super-spreading social events for COVID-19 transmission: evidence from the investigation of six early clusters in Bahrain

**DOI:** 10.3389/fpubh.2023.1216113

**Published:** 2023-09-07

**Authors:** Adel Alsayyad, Sadok Chlif, Afaf Mohamed, Fatema Habbash, Zahra Ayoob, Amer Almarabheh, Kubra Al Sayed, Aseel Alsaleh, Maryam Alhajeri, Salman Alzayani, Najat Abu Alfatah, Jamil Ahmed, Afif Ben Salah

**Affiliations:** ^1^Ministry of Health, Manama, Bahrain; ^2^Family and Community Medicine Department, College of Medicine and Medical Sciences, Arabian Gulf University, Manama, Bahrain; ^3^Institut Pasteur de Tunis, Tunis, Tunisia; ^4^King Abdullah University Medical Centre, Manama, Bahrain

**Keywords:** COVID-19, clusters, incubation period, serial interval, social events, Bahrain

## Abstract

**Introduction:**

This study aimed to characterize six early clusters of COVID-19 and derive key transmission parameters from confirmed cases that were traced between April and June 2020 in Bahrain.

**Methods:**

Pairs of “infector-infectee” allowed us to map the clusters and estimate the incubation period serial interval as the secondary attack rate. The chi-squared test, with a *p*-value computed using the Monte Carlo test, measured associations between categorical variables. Statistical analysis was performed using R software and the “data.tree, tidyverse” libraries.

**Results:**

From 9 April to 27 June 2020, we investigated 596 individuals suspected of COVID-19, of whom 127 positive cases were confirmed by PCR and linked in six clusters. The mean age was 30.34 years (S.D. = 17.84 years). The male-to-female ratio was 0.87 (276/318), and most of the contacts were of Bahraini citizenship (511/591 = 86.5%). Exposure occurred within the family in 74.3% (411/553), and 18.9% of clusters' cases were symptomatic (23/122 = 18.9%). Mapped clusters and generations increased after 24 May 2020, corresponding to “Aid El-Fitr.” The mean incubation period was 4 days, and the mean serial interval ranged from 3 to 3.31 days. The secondary attack rate was 0.21 (95% C.I.) = [0.17–0.24].

**Conclusion:**

COVID-19 transmission was amplified due to the high number of families mixing during “Aid El Fitr” and “Ramadhan,” generating important clusters. Estimated serial intervals and incubation periods support asymptomatic transmission.

## 1. Introduction

The first positive COVID-19 case in Bahrain was a 26-year-old male school car driver diagnosed on 24 February 2020. The patient presented with a fever, cough, and a travel history to a neighboring country within 14 days prior to the onset of symptoms. Since then, the number of positive cases has increased exponentially, reaching more than 200,000 by June 2020 and 375,339 after 2 years.

Preparedness for the pandemic started early in Bahrain with the nomination of a COVID-19 national response committee involving all stakeholders to conceive and implement a national plan to control the spread of the infection and reduce the burden of the pandemic (1, 2). Cases were confirmed based on the positive result of the reverse transcription polymerase chain reaction test (RT-PCR). Indeed, RT-PCR screening has been implemented at the airport of Bahrain for travelers arriving from China since the earlier phase of the pandemic (3) and has gradually included passengers from other destinations following the worldwide expansion of the pandemic. All the positive cases are reported to an electronic database from the laboratories to the public health department for timely analysis and control. Contact tracing and isolation were two main pillars of the response plan in Bahrain. The process of contact tracing survey and isolation was initiated for eligible contacts upon receiving notification of an index case, following the guidelines set forth by the WHO (2). This strategy is pivotal to controlling the spread of COVID-19 (3–5) and enables the monitoring of the transmission dynamics of the virus and the spectrum of clinical manifestations (6, 7).

Despite the early implementation of this comprehensive response plan, clusters continued to emerge in Bahrain. Similar to most of the Gulf Cooperation Countries (GCC), Bahrain is characterized by a mixed population with a high proportion of expatriates with different demographic characteristics (young population and more men) and sociocultural backgrounds; this heterogeneity, as well as differences in mixing patterns, complicate the dynamics and features of disease spread. The fasting month of “Ramadhan,” crowned by the “Aid El-Fitr,” is a socio-cultural event that causes high mixing between families and exacerbates the transmission of COVID-19. The importance of such events for the increase in transmission and cluster generation is not well-documented. In this context, SARS-CoV-2 transmission parameters, such as the latent period and the serial interval, are not estimated, which could compromise the effectiveness of predictive models and the performance of response plans. The present study aimed to elucidate transmission scenarios and characterize the patterns of COVID-19 clusters before and after “Aid El-Fitr.” We also aimed to derive the transmission parameters of the SARS-CoV-2 virus from field data. The findings will allow the adaptation of the response plans to sociocultural events that are similar to those in other countries in the region.

## 2. Methods

### 2.1. Study subjects

The study sample included all confirmed COVID-19 cases reported to the public health directorate of Bahrain following a contact tracing survey between April and June 2020.

### 2.2. Study variables

The variables analyzed in this study included age, gender, nationality (Bahrain vs. expatriate), date of the positive RT-PCR test, relatedness to the index case, the context of the transmission (in the family, in the workplace, in healthcare settings, or elsewhere), and the presence of symptoms.

### 2.3. Case definitions

The incubation period and serial interval are epidemiological parameters that describe the dynamics of transmission and disease within the population. They are defined as follows: The incubation period was the time interval between exposure and the appearance of the first symptoms in a case. The serial interval was the time between the onset of symptoms in a primary case-patient (the infector) and the onset of symptoms in a secondary case-patient (the infectee) with symptom onset (8, 9).

The secondary attack rate was defined by the number of positive cases occurring among household contacts during the incubation period post-exposure to the primary case (10).

### 2.4. Statistical analysis and computation of clusters

The cluster dataset was transformed from tabular format to tree format. The tree data structure allowed for the computation of the following variables: (i) the number of contacts made by a patient, (ii) the number of positive contacts made by a patient, and (iii) the date of the PCR result of the infector.

Pearson's chi-squared test using (i) an asymptotic chi-squared distribution to test differences in attack rates between clusters and (ii) a Monte Carlo simulation (2,000 replicas) tested the significance of differences in attack rates between generations. The initial dataset was managed using Excel [Microsoft Excel for Microsoft 365 MSO (Version 2208 Build 16.0.15601.20590 64-bit)]. Statistical analysis was performed using the R Software [R version 4.2.3—(C) developed by the R Foundation for Statistical Computing. The analysis involved employing the Platform x86_64-w64-mingw32/x64] and the “data.tree, tidyverse” libraries.

### 2.5. Ethical and regulatory considerations

The public health directorate provided the anonymized data following approval from the supreme health authorities in Bahrain. No ethical approval was required for the analysis of surveillance data in Bahrain.

## 3. Results

### 3.1. Description of the investigated sample

From 9 April to 27 June 2020, the contact tracing team from the public health directorate investigated a total of 596 suspected COVID-19 cases, of whom 127 were confirmed through PCR testing, and this included six index cases. The mean age of individuals was 30.34 years [with a minimum of 0 years (<12 months) and a maximum of 90 years, with an SD of 17.84 years]. The sex ratio was 0.87 (276/318), and most of the investigated contacts were of Bahraini citizenship (511/591 = 86.5%). Interestingly, exposure to transmission occurred mainly within the family, accounting for 74.3% of cases (411/553). However, the workplace and healthcare settings were identified as sources of transmission in 15.6 and 9.0% of cases, respectively. Infected individuals (*n* = 127) were mostly Bahrain nationals, and most infected exposure occurred in families. Most of the positive cases in the clusters were asymptomatic (23/122 = 18.9%).

The six clusters labeled from A to F are described below to provide a detailed insight into transmission timelines and context, including relatedness and mixing patterns among the infected cases.

### 3.2. Cluster A: April 2020

The index case (A001), a 24-year-old Bahraini man, developed a fever and shortness of breath on 9 April 2020 and tested positive for COVID-19. His close contacts at home and at work were identified and tested. After 2 days, one of his asymptomatic coworkers tested positive (A003), followed by his wife (A006) and her mother (A033), who tested positive 1 and 2 days later, respectively, and both were asymptomatic. After 3 days, a 21-year-old woman (A024), sister of A003, initially tested negative during the first contact tracing, developed a cough and shortness of breath, and tested positive.

Similarly, on 17 April 2020, Case A011, a 29-year-old Syrian woman and a relative of Case A003, was in isolation. Initially, she tested negative but later developed body aches; a subsequent PCR test confirmed that she was positive for COVID-19. The following day, Case A007, a 1-year-old girl and the daughter of Case A003, also showed symptoms of cough and fever while in isolation with her family. A second PCR test confirmed her positive diagnosis. To summarize, Case A003 had been in contact with 55 people during family gatherings; of these, five subsequently tested positive and are considered second-generation contacts.

### 3.3. Cluster B: May 2020

The index Case B001, a 40-year-old symptomatic man, who developed cough and fever), tested positive for COVID-19 on 29 April 2020. Contact tracing revealed 76 contacts. Upon initial testing, 16 asymptomatic family members tested positive (B002–B017). Four additional asymptomatic family members tested positive during the self-isolation exit test (B018–B021). In total, the cases (B001) were mixed with 76 traced cases. Among them, 20 were confirmed to be positive contacts.

Cases B101 and B 102 were both asymptomatic positive cases that resulted from secondary transmissions of Case B008. Case B007 had 20 close contacts, of whom her mother tested positive (B099) without exhibiting any symptoms.

Though asymptomatic during the isolation period, Case B018 led to the emergence of two secondary positive asymptomatic cases, namely Cases B146 and B147. Case B088, a 42-year-old Bahraini man, was a second-generation contact of Case B006. Despite initially testing negative on PCR, he developed a cough and runny nose, eventually testing positive 12 days later. Notably, he did not report any interactions with other positive cases during this 12-day period. The contact tracing process for Case B088 led to the identification of third-generation positive contacts, Cases B166 and B167, both of whom were his family members and were asymptomatic (Table 1).

**Table 1:** Profile of the study sample and infected individuals.

	**Total study sample (*****n*** = **596)**		**Infected individuals (*****n*** = **127)**		
	***N***	**(%)**	***N***	**(%)**	**Prevalence (%)**
**Age**					
0–20	157	(27.8)	50	(40.7)	(31.8)
20–40	233	(41.3)	37	(30.0)	(15.9)
40–60	143	(25.4)	27	(22.0)	(18.9)
60+	31	(5.5)	9	(7.3)	(29.0)
Total	564	(100.0)	123	(100)	(21.8)
**Gender**					
Men	276	(46.5)	56	(44.1)	(20.3)
Women	318	(53.5)	71	(55.9)	(22.3)
Total	594	(100)	127	(100)	(21.4)
**Nationality**					
Bahraini	511	(86.5)	122	(96.1)	(23.9)
Non-Bahraini	80	(13.5)	5	(3.9)	(6.3)
Total	591	(100)	127	(100)	(21.4)
**Presence of symptoms**					
Yes			23	(18.9)	
No			99	(81.1)	
Total			122	(100)	
**Place of exposure**					
Work	86	(15.6)	2	(2.2)	(2.3)
Family	411	(74.3)	89	(95.6)	(21.7)
Clinic	50	(9.0)	0	(0.0)	(0.0)
Other places	6	(1.1)	2	(2.2)	(33.3)
Total	553	(100)	93	(100)	(16.8)

### 3.4. Cluster C: May 2020

The index case (C001), a 33-year-old Bahraini male healthcare worker, tested positive on 29 May 2020, without exhibiting any symptoms. Within the next 3 days, he came into contact with 23 individuals. Among these contacts, 11 of his family members tested positive on the PCR test (C002–C012). They were all asymptomatic except for cases C004 and C011, who both complained of a dry cough. Case C007 had six close contacts in the second generation, all of whom were subjected to testing. Among these contacts, only one individual, identified as CO50, tested positive. Notably, this 25-year-old Bahraini woman displayed no symptoms. During the contact tracing for Case C009, 17 individuals were identified as second-generation contacts and tested. Among them, eight family members (C054–C061) showed a positive PCR result on the first swab test despite being asymptomatic. In addition, another asymptomatic family relative (C062) tested positive during the exit isolation PCR test conducted on 11 June 2020.

Case (C055), a 40-year-old woman, had six contacts (third generation) who were tested. One tested positive with no symptoms (C127). Similarly, cases C112 and C113, both of whom are relatives of Case C054, tested positive through PCR while in isolation despite having initially tested negative during the contact tracing process.

### 3.5. Cluster D: June 2020

The index case (D001), a 34-year-old Bahraini man, tested positive, though asymptomatic, during a random “Drive Thru” testing on 5 May 2020. Contact tracing revealed that 19 of his family members tested positive for COVID-19, including his 28-year-old wife (D002), within 1–3 days. Four of his family contacts tested positive. Two of them were symptomatic and tested positive since the first PCR test (D012) and (D018). The two other cases, D019 and D020, developed symptoms (sore throat and runny nose) 2 days after the initial negative swab test and subsequently tested positive through PCR. In the case of Case D002, there were four contacts in the second generation, of whom only one tested positive with no symptoms [her son (D021), a 3-year-old male].

### 3.6. Cluster E: June 2020

Case (E001), a 49-year-old Bahraini man, complained of fever, cough, and a runny nose. He did not report any recent travel or contact with positive cases. He tested positive for COVID-19 on 7 May 2020. All of his close contacts were eight family members, of whom five were positive with no symptoms (E002–E006).

His relative (E006) had eight contacts in the second generation who were also family members. Among them, five tested positive (E010–E014). The contact tracing of Case E011 at the workplace revealed an asymptomatic positive case in the third generation (E032). The latter case (E032) was a 37-year-old Bahraini man with 22 contacts as the fourth generation, of whom 10 tested positive and were all family members (E033–E042). Among these 10 positive contacts, five complained of sore throats, and one had a loss of smell.

### 3.7. Cluster F: June 2020

Index case F001, a 37-year-old Bahraini woman, was asymptomatic upon testing positive for COVID-19 on June 11 2020. Contact tracing among 44 contacts revealed 13 positive cases (F002–F011) and (F013–F015), who were all family members. Among them, three individuals exhibited symptoms: Case F005 presented with a cough and sore throat, Case F008 displayed symptoms of fever, cough, and sore throat necessitating hospitalization, and Case F011 reported Among them, three individuals exhibited symptoms: Case F005 presented with a cough and sore throat, Case F008 displayed symptoms of fever, cough, and sore throat necessitating hospitalization, and Case F011 reported fever, sore throat, and fatigue.

Case F002, a 73-year-old woman, had 14 contacts, of whom two tested positive: Case F (034) and Case F035. Initial contact tracing of Case F011 resulted in two positive cases: Case F (056) and Case F057. Case F058, placed in isolation, showed a positive result 12 days later (25 June 2020) at the exit swab sample. The five cases formed the second generation. The transmission chain continued as Case F035 developed symptoms of cough, sore throat, and fatigue and caused two other positive cases, Cases F048 and F012. Case F058 resulted in further transmission of the infection in Case F059 2 days later. The three latter cases of the third generation did not exhibit any symptoms.

**Figure 1:**
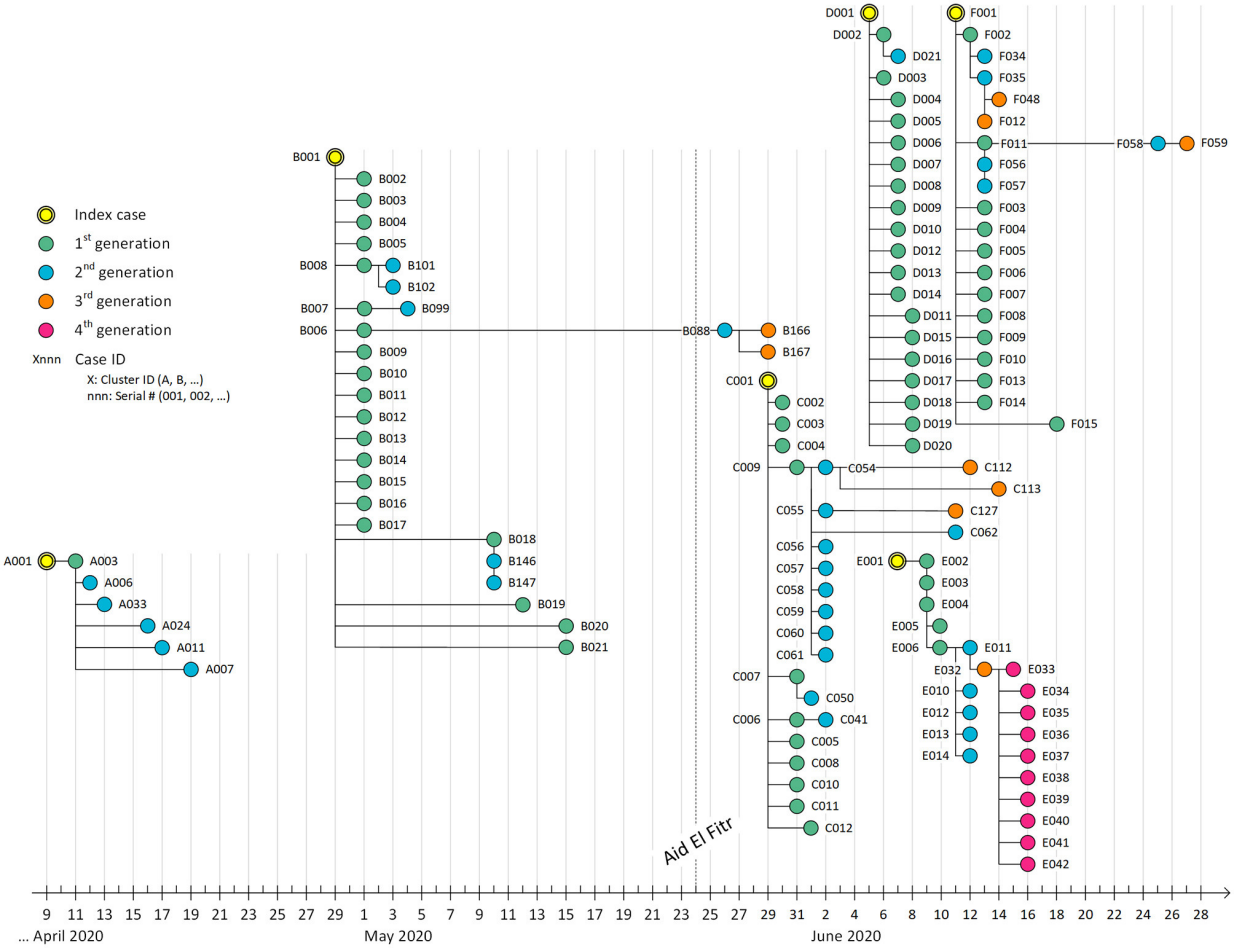
Transmission map of COVID-19 cases detected during active contact tracing between 9 April 2020 and 28 June 2020. Each bullet represents a PCR+ case. The location on the X-axis represents the date of the PCR positive result. Each edge represents a positive contact. We did not include negative cases in this map to ease visual interpretation. After Aid El-Fitr (24 May 2020), the number of clusters increased much faster (~4 clusters per month) compared to the period between the start of the epidemic and Aid El-Fitr (~1 cluster per month).

The mapping of the COVID-19 clusters and related generations over time is detailed in Figure 1. It indicates that transmission was relatively slow during the start of the pandemic in Bahrain in the month of April, as shown in the first cluster. Following the fasting month of “Ramadhan” in Bahrain and the celebration of “Aid El-Fitr” on 24 May, transmission was exacerbated due to high mixing within families during this religious event, significantly increasing infected individuals, clusters, and generations.

The incubation period was estimated from data (*n* = 13 cases) as the duration between the last day of exposure to the infector and the date of sampling for COVID-19 in a confirmed positive “infectee” who is symptomatic. It ranged from 1 to 9 days (mean = 4 days, 95% CI of the mean = [2.00, 6.00], S.D. =3.31 and median= 2 days, [Q1 = 2, Q3 = 7]).

Unfortunately, the symptom status for pairs of “infector-infectee” was not available for all pairs in our data. This limitation caused the need to estimate the serial interval in two ways.

First, the serial interval was estimated from pairs of “infector-infectee” by determining the duration in days between the date of sampling of the positive “infector,” confirmed by PCR, without considering the presence of symptoms, and the date of sampling of the related symptomatic “infectee,” also confirmed by PCR. The range for this interval spanned from 1 to 8 days [mean = 3 days, 95% CI of the mean = [2.20, 3.80], S.D.= 1.72 days, and median = 3 days [Q1 = 2, Q3 = 4], (*n* = 20 pairs)].

Second, the serial interval was estimated from pairs of “infector-infectee” as the duration in days between the date of sampling of a symptomatic infector and the sampling date of “infectee” disregarding symptoms. It ranged from 1 to 15 days [mean = 3.31days, 95% CI of the mean = [1.57, 5.05], S.D. =4.58 days, and median = 1 day, [Q1 = 1, Q3 = 2], (*n* = 29 pairs)].

Interestingly, for both estimations of the serial interval, the mean was ~3 days. However, the median was slightly higher (4 days) in the first estimation while it was overdispersed, leading to a lower median (1 day) in the second estimation.

**Figure 2:**
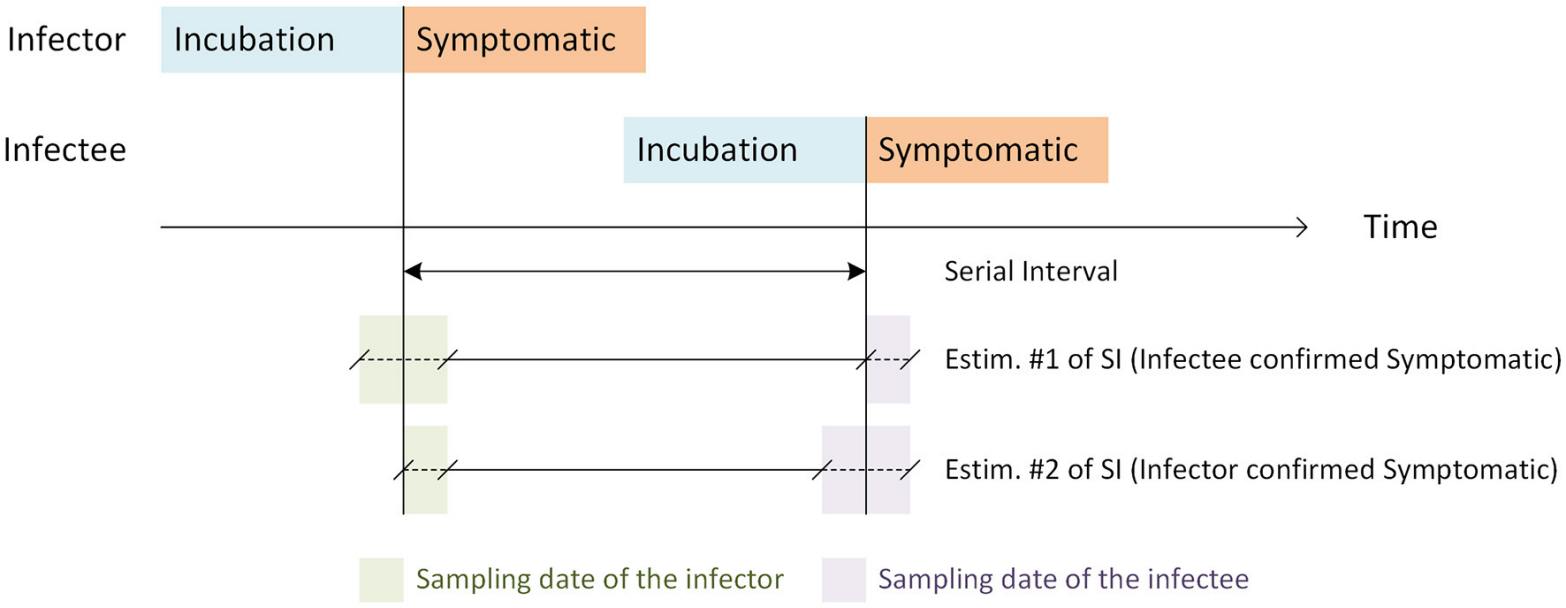
Graphical illustration showing the definition and estimation methods of the serial interval. For estimation #1, the symptomatic status is confirmed for the infectee and unknown for the infector, and for estimation #2, the symptomatic status is confirmed for the infector and unknown for the infectee. The top double arrow line represents the serial interval when the symptomatic status is known for both the infector and the infectee.

Figure 2 clarifies the approach for estimating the serial interval given the constraints of the unavailability of symptom status for the pairs of “infector-infectee” in our data set.

The secondary attack rate was 0.21 with 95% confidence intervals (95% CIs) = [0.17–0.24] within six clusters and five generations. This rate was 0.45 (69 cases/153 contacts) when considering the first generation at 95% CIs [0.370–0.533] and dropped to 0.14 (33 cases/242 contacts) at 95% CIs [0.095–0.186] in the second generation.

The proportion of the infected patients among contacts revealed an overdispersed distribution according to the generations where the most infections occurred during the first generation (Pearson's Chi-squared test with *p*-value computed using the Monte Carlo test, which was χ^2^ = 95.006, *p*-value = 0.0004998 <10^−3^ (11).

However, most of the cases detected are in the last three clusters, D, E, and F, where the proportion infected per cluster showed a significant steady increase over time (Pearson's χ^2^ test = 48.098, df = 5, *p*-value = 3.392 10^−9^ <10^−3^) (Table 2).

**Table 2:** Attack rates among clusters and generations.

**Cluster**	**Generation**												
	**1st gen**.		**2nd gen**.		**3rd gen**.		**4th gen**.		**5th gen**.		**Total**		**Attack rate** ^*^
	**Tested**	**PCR**+**(%)**	**Tested**	**PCR**+**(%)**	**Tested**	**PCR**+**(%)**	**Tested**	**PCR**+**(%)**	**Tested**	**PCR**+**(%)**	**Tested**	**PCR**+**(%)**	
A	3	1 (33)	55	5 (9)	55	0 (0)	0	0	0	0	113	6 (5)	0.05 [0.02–0.11]
B	76	20 (26)	74	6 (8)	15	2 (13)	0	0	0	0	165	28 (17)	0.17 [0.12–0.24]
C	23	11 (48)	54	11 (20)	63	3 (5)	0	0	0	0	140	25 (18)	0.18 [0.12–0.25]
D	19	19 (100)	23	1 (4)	0	0	0	0	0	0	42	20 (48)	0.48 [0.32–0.64]
E	8	5 (63)	12	5 (42)	11	1 (9)	22	10 (45)	15	0 (0)	68	21 (31)	0.31 [0.20–0.43]
F	24	13 (54)	24	5 (21)	12	3 (25)	2	0 (0)	0	0	62	21 (34)	0.34 [0.22–0.47]
Total	153	69 (45)	242	33 (14)	156	9 (6)	24	10 (42)	15	0 (0)	590	121 (21)	
Attack rate^*^	0.45 [0.37–0.53]	0.14 [0.09–0.19]	0.06 [0.03–0.11]	0.42 [0.22–0.63]	0.00 [0.00–0.22]	0.21 [0.17–0.24]	

^*^Attack rate [95% C.I].

The first column identifies the six clusters from (A to F) and the second row represents the five generations computed from all clusters.

The last column shows the attack rates with 95% confidence limits for the six clusters, and the last row shows the attack rates with 95% confidence limits per generation and overall.

## 4. Discussion

This study comprehensively analyzed 129 cases in six COVID-19 clusters resulting from the active screening of 590 contacts. It allowed the accurate identification of the timeline for the spread of the SARS-CoV-2 virus in the sociocultural context of Bahrain for the first time, which is similar to other GCC countries. Mixing information about the context of transmission, the symptoms, the relatedness of cases, and the date of positive PCR clarified the transmission scenarios and permitted the estimate of key epidemiological parameters from field data. This information is useful to conceive realistic models to forecast the course of the pandemic in the population's specific context and to refine future control strategies.

The COVID-19 response team of Bahrain recommended the policy of actively testing by PCR, tracing, isolating, and treating as an important pillar to curb the pandemic in its early stages, which is in agreement with the WHO recommendations and the guidelines of other countries (12). This approach helped identify the source of COVID-19 infections and the rapid containment of transmission and severity. Although the gold standard, this test is limited by its ability to detect cases during the period of viral shedding, as it could be falsely negative among those tested at earlier stages of contact with cases (12).

The clusters under study were identified from April to June 2020, 2 months after diagnosing the first case in the Kingdom of Bahrain. They summarize valuable information about the epidemiological course of the COVID-19 pandemic in this country in its early stages. The cases under study were mainly due to the local transmission of the disease in the country, reflecting the mixing patterns in the population despite the imposed restrictive control measures. This finding was confirmed in Qatar as well (13). The mapping of clusters through time showed that “Aid El Fitr,” a religious event Muslims celebrate to mark the end of the fasting in the month of “Ramadhan” that occurred on 24 May 2020, amplified transmission through high mixing within families. We also noticed the high clustering of cases over time, as shown in Figure 1. However, most cases were discovered during the first generation, reflecting the increased awareness following contact tracing and the performance of the response team in interrupting transmission through various preventive measures recommended by the national response team of Bahrain.

Most of the contacts identified in the clusters and most of the positive cases were among family contacts (21% or 89/411 contacts). This pattern is not surprising given the proximity and prolonged exposure to the index case and the routes of transmission through droplet and aerosol particles (14). Contacts with positive cases in other places are also at risk of disease transmission, especially with prolonged interaction and high mixing (3). The COVID-19 virus was found to be transmitted in different settings; however, many of the described clusters happened indoors among household contacts, with a small number of positive cases (15). Several other factors, such as the viral load, the airflow in the place, and other environmental conditions, determined the probability of transmission (16). The transmission events, measured as PCR positivity at any time up to 14 days post-exposure (14), appeared to be independent of age and gender (17).

The incubation period (IP) and serial intervals (SI) were estimated from the data in the present study. The mean SI estimated from the data by two approximations, depending on the availability of information about the symptom's status in one of the elements of pairs (infector-infectee), ranged from 3 to 3.31 days. A more accurate estimation lies between the two estimations, which agrees with that reported in a previous study conducted in Brazil (18). However, the mean IP was 4 days. Despite the reduced sample size used for their estimation, both IP and SI were in the range reported in a review of 19 studies published between January 2020 and 10 March 2022 (19). Our estimates of SI and IP suggested that SI is shorter than IP, which is in agreement with the asymptomatic transmission of the SARS-CoV-2 virus.

The secondary attack rate observed among contacts within the Bahrain clusters was 21%, with 95% CIs of 17–24%, amounting to 121 out of 590 contacts. This rate aligns with findings from other studies, where estimations ranged between 4.6 and 49.6%. Notably, the secondary attack rate did not exhibit any correlation with the geographical region or the imposition of lockdown measures (10, 14). Notably, household transmission contributed to the spread of COVID-19 cases among the most vulnerable groups in countries where national lockdowns and social distancing were imposed. Indeed, the variation in the attack rate resulted from the high mixing between susceptible and infectious individuals and because of the differences in the viral load among the infected. These transmission scenarios are consistent with those reported by other studies from most other countries during the early months of the pandemic. These studies also show that close family members were the most likely contacts to become infected (17). However, in early clusters in the United States, family gatherings were also found to be a source of the spread of SARS-CoV-2 outside the household (20).

Patients with COVID-19 might be unable to report their symptoms, especially when they suffer from a mild illness. In addition, in family clusters, the patients may fail to report their symptoms or may be asymptomatic, and hence, they neither isolate from other family members nor seek medical advice. These infectious cases may transmit the virus to their contacts, a situation in which diagnosis may be missed (3, 21). Most COVID-19 patients were asymptomatic in Bahrain and Qatar, subsequently acting as a pool for virus transmission and creating new clusters of disease (22, 23). This pattern was confirmed by our study, where 81% (99/122) of the PCR-confirmed cases were asymptomatic. This proportion was higher than that previously reported, where it was found that 50% of the investigated cases were asymptomatic (24). This high proportion of asymptomatic positive cases observed in our study could reflect the effective performance of the contact tracing process and the response team's efforts upon receiving notifications about index cases. Additionally, it could reflect the impact of the isolation and testing policy for both cases and their associated contacts. Assuming the risk of transmission even among negative contacts, all those who tested negative around an index case during the contact tracing survey in Bahrain were isolated for 14 days and tested before discontinuing isolation, even if they remained asymptomatic. However, if any of the contacts developed symptoms during the isolation period, they received the second PCR test. This policy of testing the contact twice allowed the early detection of many positive cases, even if they were asymptomatic, thus reducing the viral shedding duration, which was found to be similar among the symptomatic and the asymptomatic positive cases in Bahrain (22).

## 5. Conclusion

This study confirmed that mixing between families during religious and social events remained an important driver of transmission in Bahrain despite the impressive response of the national team, the rapid implementation of effective non-pharmaceutical control measures, and the availability of anti-COVID-19 vaccination through involvement in vaccine trials during the early stages of the pandemic. Mixing during sociocultural events was the least compressible risk in the control strategy of COVID-19 in Bahrain and other GCC countries. Our findings support the importance of considering social and cultural determinants of transmission in future pandemic response plans. Overall, the control of the COVID-19 pandemic in Bahrain was one of the most successful worldwide, with limited morbidity and mortality loads and minor impacts on the performance of the curative and preventive services of the health system.

## Data availability statement

The raw data supporting the conclusions of this article will be made available by the authors, without undue reservation.

## Ethics statement

Ethical approval was not required for the study involving humans in accordance with the local legislation and institutional requirements. Written informed consent to participate in this study was not required from the participants or the participants' legal guardians/next of kin in accordance with the national legislation and the institutional requirements.

## Author contributions

AAlsay, SC, FH, NA, and MA developed the protocols. AAlsay, SC, AM, ZA, KA, AAlsal, SA, JA, and AB reviewed the cluster data. SC performed the data management and production of the clusters' maps. AB, SC, and AAlm performed statistical analysis.
