# Supplementary material for: Super-spreading social events for COVID-19 transmission: evidence from the investigation of six early clusters in Bahrain
Source: Front Public Health. 2023 Sep 7;11:1216113. doi: 10.3389/fpubh.2023.1216113 (PMC10513455; doi:10.3389/fpubh.2023.1216113)
Supplement: Supplementary file 1 [file Data_Sheet_1.PDF]

**Dataset#1 :**

Dataset used to estimate the serial interval based on the first method (n=20 pairs).

| # | Cluster | Pair (Infector, Infectee) | Infectee sampling date | Infectee PCR test result | Infectee presence of symptoms | Infector sampling date | Infector PCR test result | Infector presence of symptoms | Serial interval (days) |
|---|---|---|---|---|---|---|---|---|---|
| 1 | A | (A003, A007) | 2020-04-18 | Positive | Yes | 2020-04-10 | Positive | No | 8 |
| 2 | A | (A003, A024) | 2020-04-16 | Positive | Yes | 2020-04-10 | Positive | No | 6 |
| 3 | C | (C001, C003) | 2020-05-29 | Positive | Yes | 2020-05-28 | Positive | | 1 |
| 4 | C | (C001, C010) | 2020-05-31 | Positive | Yes | 2020-05-28 | Positive | | 3 |
| 5 | D | (D001, D010) | 2020-06-07 | Positive | Yes | 2020-06-04 | Positive | No | 3 |
| 6 | D | (D001, D016) | 2020-06-08 | Positive | Yes | 2020-06-04 | Positive | No | 4 |
| 7 | D | (D001, D017) | 2020-06-08 | Positive | Yes | 2020-06-04 | Positive | No | 4 |
| 8 | D | (D001, D018) | 2020-06-08 | Positive | Yes | 2020-06-04 | Positive | No | 4 |
| 9 | D | (D001, D019) | 2020-06-08 | Positive | Yes | 2020-06-04 | Positive | No | 4 |
| 10 | E | (E032, E033) | 2020-06-15 | Positive | Yes | 2020-06-13 | Positive | No | 2 |
| 11 | E | (E032, E036) | 2020-06-15 | Positive | Yes | 2020-06-13 | Positive | No | 2 |
| 12 | E | (E032, E038) | 2020-06-15 | Positive | Yes | 2020-06-13 | Positive | No | 2 |
| 13 | E | (E032, E040) | 2020-06-15 | Positive | Yes | 2020-06-13 | Positive | No | 2 |
| 14 | E | (E032, E041) | 2020-06-15 | Positive | Yes | 2020-06-13 | Positive | No | 2 |
| 15 | E | (E032, E042) | 2020-06-15 | Positive | Yes | 2020-06-13 | Positive | No | 2 |
| 16 | F | (F002, F035) | 2020-06-12 | Positive | Yes | 2020-06-11 | Positive | No | 1 |
| 17 | F | (F035, F012) | 2020-06-13 | Positive | Yes | 2020-06-12 | Positive | Yes | 1 |
| 18 | F | (F001, F005) | 2020-06-13 | Positive | Yes | 2020-06-10 | Positive | No | 3 |
| 19 | F | (F001, F008) | 2020-06-13 | Positive | Yes | 2020-06-10 | Positive | No | 3 |
| 20 | F | (F001, F011) | 2020-06-13 | Positive | Yes | 2020-06-10 | Positive | No | 3 |

**Dataset#2:** (29 pairs)
Dataset used to estimate the serial interval based on the second method (n=29).

| # | Cluster | Pair (Infector, Infectee) | Infectee sampling date | Infectee PCR test result | Infectee presence of symptoms | Infector sampling date | Infector PCR test result | Infector presence of symptoms | Serial interval (days) |
|---|---|---|---|---|---|---|---|---|---|
| 1 | A | (A001, A003) | 2020-04-10 | Positive | No | 2020-04-08 | Positive | Yes | 2 |
| 2 | B | (B001, B002) | 2020-04-30 | Positive | No | 2020-04-29 | Positive | Yes | 1 |
| 3 | B | (B001, B003) | 2020-04-30 | Positive | No | 2020-04-29 | Positive | Yes | 1 |
| 4 | B | (B001, B004) | 2020-04-30 | Positive | No | 2020-04-29 | Positive | Yes | 1 |
| 5 | B | (B001, B005) | 2020-04-30 | Positive | No | 2020-04-29 | Positive | Yes | 1 |
| 6 | B | (B001, B006) | 2020-04-30 | Positive | No | 2020-04-29 | Positive | Yes | 1 |
| 7 | B | (B001, B007) | 2020-04-30 | Positive | No | 2020-04-29 | Positive | Yes | 1 |
| 8 | B | (B001, B008) | 2020-04-30 | Positive | No | 2020-04-29 | Positive | Yes | 1 |
| 9 | B | (B001, B009) | 2020-04-30 | Positive | No | 2020-04-29 | Positive | Yes | 1 |
| 10 | B | (B001, B010) | 2020-04-30 | Positive | No | 2020-04-29 | Positive | Yes | 1 |
| 11 | B | (B001, B011) | 2020-04-30 | Positive | No | 2020-04-29 | Positive | Yes | 1 |
| 12 | B | (B001, B012) | 2020-04-30 | Positive | No | 2020-04-29 | Positive | Yes | 1 |
| 13 | B | (B001, B013) | 2020-04-30 | Positive | No | 2020-04-29 | Positive | Yes | 1 |
| 14 | B | (B001, B014) | 2020-04-30 | Positive | No | 2020-04-29 | Positive | Yes | 1 |
| 15 | B | (B001, B015) | 2020-04-30 | Positive | No | 2020-04-29 | Positive | Yes | 1 |
| 16 | B | (B001, B016) | 2020-04-30 | Positive | No | 2020-04-29 | Positive | Yes | 1 |
| 17 | B | (B001, B017) | 2020-04-30 | Positive | No | 2020-04-29 | Positive | Yes | 1 |
| 18 | B | (B001, B018) | 2020-05-10 | Positive | No | 2020-04-29 | Positive | Yes | 11 |
| 19 | B | (B001, B019) | 2020-05-12 | Positive | No | 2020-04-29 | Positive | Yes | 13 |
| 20 | B | (B001, B020) | 2020-05-14 | Positive | No | 2020-04-29 | Positive | Yes | 15 |
| 21 | B | (B001, B021) | 2020-05-14 | Positive | No | 2020-04-29 | Positive | Yes | 15 |
| 22 | E | (E001, E002) | 2020-06-09 | Positive | No | 2020-06-07 | Positive | Yes | 2 |
| 23 | E | (E001, E003) | 2020-06-09 | Positive | No | 2020-06-07 | Positive | Yes | 2 |
| 24 | E | (E001, E004) | 2020-06-09 | Positive | No | 2020-06-07 | Positive | Yes | 2 |
| 25 | E | (E001, E005) | 2020-06-09 | Positive | No | 2020-06-07 | Positive | Yes | 2 |
| 26 | E | (E001, E006) | 2020-06-09 | Positive | No | 2020-06-07 | Positive | Yes | 2 |
| 27 | F | (F035, F012) | 2020-06-13 | Positive | Yes | 2020-06-12 | Positive | Yes | 1 |
| 28 | F | (F035, F048) | 2020-06-14 | Positive | No | 2020-06-12 | Positive | Yes | 2 |
| 29 | F | (F011, F058) | 2020-06-24 | Positive | No | 2020-06-13 | Positive | Yes | 11 |
